# Prof. Arneman, on Acoustic Tubes

**Published:** 1800-10

**Authors:** 


### *Prof. Arneman, on Acoustic Tubes.*

[ With an Engraving. ]

The ufe of acouftic tubes, (hearing trumpets) depends on two points; 1. That the found is put into a ftronger vibrating motion through the medium of the metal, and at the fame time; 2. That a refonance, and new reflection of the found, is produced.

The found increafes and becomes ftronger by thefe inftruments, but if they are not properly conftructed, it is likewife made lefs diftinguifhable. The beft acouftic tubes are the moft fimple ones, either quite ftraight, or with a little curvity. It is alfo effential, they fhould have a wide opening to receive a greater portion of found, and by which it is brought at the fame time more condenfed to the organs of hearing. Thofe that are much curved, have no great effect, and likewife the inconvenience that on account of their curved line they cannot be worked fo even by the artift. The fame may be faid of the conoidical tubes, in which the found ceafes to be reflected at the place where the angle grows greater than a right one, and becomes retrograde; becaufe, according to Lambert's Obfervations, (on Acouftic Inftruments, in German) the angles of reflection increafe like unequal numbers; and the laft which can be admitted, muft not exceed 90°. The form of an entire cone, is therefore by no means calculated for propagating the found, which, having entered, goes out again, without reaching the top of the cone. The parabolic form is the fitteft for the conftruction of acouftic tubes, into which the found enters in a direction parallel with the axis, and is by reflection concentrated as it were in a focus. Acouftic inftruments with an hemifphere to them, are inferior to thefe, becaufe the found is only reflected from the centre, and at the fame time not fo eafily intercepted.

However, it feems that the conftruction of thefe inftruments has not been brought to fo great a perfection, as not to admit fome improvements founded upon practice and experience. For the purpofe of promoting this, Mr. Arneman intends to give, by degrees, figures and defcriptions of thofe that have proved moft ufeful by experience, and he invites all practitioners in Germany, who have had an opportunity of making obfervations to this fubject, to communicate any inftrument of the kind they have applied with fuccefs, by which means much would be contributed to a more perfect and fure method of diminifhing thofe inconveniences arifing from difficult hearing, for which fo many feek advice and relief in vain, againft an evil, which makes men unfit for enjoying life from the moft interefting mean, that of communicating and receiving ideas by word of mouth. The public would, we truft, be likewife much obliged to the Editors of this Journal, if they could perfuade the practitioners of this country, to impart their experience on this fubject here, and to contribute to fuch an ufeful and humane undertaking. As they hope not to fail in their requefts, requests, they should with pleasure receive any communication respecting the subject.

The inventor of the present instrument (see the Plate) is not known, but it belongs certainly to the best of the kind that we know of. It is made of thin brass or copper, and should not weigh more than eight ounces, whereby it becomes very portable, and may as well serve for the right as for the left ear. To enable any artist to make this instrument accurately, it has been thought proper to add the dimensions of the different parts of which it is composed. H G I F is the cup, of which H I has 3 Paris inches, 6 lines in diameter. F G = 2 Paris inches, 10 lines. H F is = 3 Paris inches, 1 line, F G is the bottom of the cup, which must particularly be made with exactness, because the use of the instrument depends in a great measure on its construction. It may be made as the segment of a circle whose radius is = 2 inches, 7 lines, Paris; but it is still better to give a parabolic form to the segment, whose focus is 1 inch Paris. For this purpose it is necessary to work after a certain model or standard, of which Fig. II. gives an idea, after which the bottom must be accurately made. The inside ought to be smooth and well polished, the whole afterwards soldered on, when the instrument is put together. The others are, that which receives the sound, D E, and the conducting tube, which carries it to the ear, K L B. The former is to be performed in the same parabolic form as the bottom of the cup, and must therefore be exactly worked after the above Fig. 2, except that its chord is by 4 or 5 Paris inches smaller than the bottom. In the middle of this part, D E, a hole is made, whose diameter is 1 Paris inch 2 ½ lines, which is at the same time the lower diameter of the tube K L, its upper diameter in A being only 6 lines. The length of the tube from K L to A, is 5 inches 10 lines. This must now be bent in such a manner, that K L and A make an angle of 90°. Being well polished inside, the part D E is soldered into it, and the whole conducting tube fastened to the cup in I L, so that D E is equally distant from all sides of the cup in an exact horizontal direction, and the focus of the part D E likewise sufficiently distant from the focus of the bottom. In A another piece of tube is put on, of 9 inches in length, and fastened to it by means of a ring on the former A, at the distance of 1 inch from its end, with an opening in its periphery, through which a hook may pass, and turning this afterwards, the tube becomes one contiguous piece. On the curvation in B, a top of bone, ivory, or horn is put, to prevent the ear being irritated and inflamed, which may otherwise be caused by the metal. The last part is the cover, H I, perforated with holes, of which Fig. III. shows the profile. The part A B being taken off, the instrument may be conveniently carried in the pocket. It can also be fastened to the body by ribbands, by means of two clinchers in M N, and thus even used in going a shooting, &c. (*Arneman's Magazine, Vol. ii. No.* 3.)

---

*Hints*

